# Breastfeeding experiences of Latina migrants living in Spain: a qualitative descriptive study

**DOI:** 10.1186/s13006-021-00423-y

**Published:** 2021-10-09

**Authors:** Blanca Iglesias-Rosado, Fatima Leon-Larios

**Affiliations:** 1 Department of Social Psychology, Psychology School, University of Seville, Seville, Spain; 2 Nursing Department, School of Nursing, Physiotherapy and Podiatry, University of Seville, Seville, Spain

**Keywords:** Barriers, Breastfeeding, Facilitators, Immigrants, Migrants, Latinas, Qualitative study

## Abstract

**Background:**

The migratory flows in Spain have changed due to the arrival of a diverse migrant population. Among the new migrants the Latino collective predominate with more than half being women of childbearing age. There are no previous studies exploring breastfeeding experiences of migrants in a country where their mother tongue is spoken. This study aimed to explore Latina migrants’ breastfeeding experiences in a Spanish-speaking country.

**Methods:**

A descriptive qualitative study was carried out in the main province in southern Andalusia between November 2019 and June 2020. The study used intentional sampling. The study participants were contacted by video calls and data were collected through a semi-structured in-depth interview (*n* = 19). The interviews were transcribed and analysed by thematic analysis.

**Results:**

The nineteen participants were aged between 22 and 43 years old and came from six different countries in Latin America. The two main categories that emerged were breastfeeding facilitators and barriers, divided into ten interrelated sub-categories: working conditions; precarious socioeconomic conditions; lack of support (health professionals, family and society); physiological changes, pain and fatigue; ignorance and wrong beliefs; support networks (partner, health professionals and family); host country versus home country; religious practices/worship; appropriate attitude, knowledge and experience; and breastfeeding support groups. Most of the study participants stated that their breastfeeding experiences were influenced by barriers such as work and by facilitators such as peer support.

**Conclusions:**

More support from caregivers and more sensitivity to cultural diversity were demanded by the women and well-trained professionals are needed to enable breastfeeding for a longer time. This paper provides caregivers, such as nurses, more knowledge about the care demanded by migrant women to ensure a longer breastfeeding experience.

**Supplementary Information:**

The online version contains supplementary material available at 10.1186/s13006-021-00423-y.

## Background

Although the World Health Organization’s (WHO) recommendations are to exclusively breastfeed up to 6 months and continue thereafter into the second year of life [1], breastfeeding rates in Spain are not as desired. According to the latest WHO report by country, there is 68.4% exclusive breastfeeding in Spain at 6 weeks postpartum which gradually decreases, reaching 24.7% at 6 months [2]. These data reflect the need for measures to promote breastfeeding in an increasingly diverse country such as Spain.

In recent years, there has been a change in the migratory flows to our country. Of the foreign population registered in Spain, 24.4% originate from Latin America, of whom 57.5% are women. The feminization of immigrants occurs at the national, regional (Andalusia), and provincial (Seville) levels. The dominant nationalities are Colombian, Venezuelan, Bolivian, Paraguayan, and Peruvian [3]. The Latin immigrant population is of childbearing age (between 20 and 39 years old) when they migrate to Spain [4]. Latina immigrants in Spain are more likely to breastfeed than Spanish women [5–8]. Previous studies have identified barriers and facilitators unique to Latina women living in non-Spanish-speaking countries [9]. Currently, there are no studies exploring the experiences of Latina women immigrants in a Spanish-speaking country [10]. Although some barriers and facilitators to breastfeed successfully are known, migrant mothers continue to face difficulties, [11–21] leading some researchers to suggest that culturally adapted health services are necessary to maintain breastfeeding rates among migrant mothers [10, 22]. The aim of this study is to explore Latina migrants’ breastfeeding experience in a Spanish-speaking country.

## Methods

### Study design

This qualitative study was carried out following the Consolidated Criteria for Reporting Qualitative Research (COREQ) that covers the reporting of studies using interviews, and was developed to promote explicit and comprehensive reporting of interviews (see Additional file 1). The study was conducted in three local districts of Seville, the main province in southern Andalusia between November 2019 and June 2020.

### Participants

Participants were selected using a non-probability “snowball” sampling procedure. The inclusion criteria were: Latina women who had given birth in Spain; experience of breastfeeding in the host country for at least 2 months over the last 5 years; involvement in breastfeeding support groups, both face-to-face or online; and consent to participate in the study. A Breastfeeding Support Group is a peer community group run by a mother with a successful experience of breastfeeding, led by a midwife. This support group provides advice on breastfeeding problems and their resolution, both online and through a private Facebook group, or during weekly face-to-face meetings. The first strategy used to recruit participants for the study was to contact the Breastfeeding Support Group on social media.

### Measures

In-depth semi-structured interviews were conducted until data saturation was reached. For this purpose, an interview script was created (see Table 1), which included three dimensions: sociodemographic factors, birth and breastfeeding history and experiences, as well as support and feelings.

**Table 1:** Interview script

Themes	Sample questions
Sociodemographic factors	What is your name? How old are you? What is your educational level? What is your country of birth? When did you arrive to Spain? Why did you migrate? What was the documentation status situation when the baby was born? Did you have maternity leave? Do you feel comfortable in Spain?
Obstetric data and knowledge	How many children do you have? How long did breastfeeding last in each one? How was your labour experience/s? Did you go to antenatal classes? In your opinion, what are breastfeeding advantages? Did you feel that you received the information you needed? Did you have difficulties for healthcare professionals to resolve your doubts? In your opinion, what are the barriers that have not helped you/other women to maintain breastfeeding for longer?
Supports and feelings	What family do you have here? Who has been your main support? Do you think that breastfeeding in your home country would have been different? What predominates in your home country: breast milk or formula? What role have healthcare professionals played in your breastfeeding experience? What do breastfeeding support groups bring you? Do you practice religion here? How did you feel/are you feeling about your breastfeeding experience? Is there anything else you would like to say?

Note. Source: Self-made

### Data collection

Due to the current public health situation caused by COVID-19 and lockdown restrictions, a total of 19 interviews were conducted online and a code was assigned (M (mother) M (migrant) L (Latina) X (number of the interview)). In this way, different video-calling applications such as Skype or WhatsApp were used and prior informed consent obtained. Under these circumstances, we took the participants’ resources into account and attempted to maintain a comfortable environment and ensure the mothers’ privacy. The interviews were conducted in Spanish by the first author, who was an MSc candidate and not involved in the care of the participants. Interviews were recorded using three different devices and transcribed by the same researcher.

### Data analysis

The interviews were analysed using an inductive content analysis, that is, a process of abstraction to reduce and group the data so that researchers could answer the study questions using concepts, categories or themes [23]. The transcribed interviews were examined using a thematic analysis to identify their meanings. For this purpose, a reflective, iterative and systematic process was performed, attending to the phases proposed by Braun and Clarke [24]. These are: 1) Familiarization with the data through readings and annotations made by both researchers; 2) Coding using a process that involved reading and rereading of the trascriptions to identify patterns and themes; 3) Preparation of a thematic mind map: codes were organized into a hierarchical structure for interpretation; 4) Defining and naming themes and subthemes; and 5) Preparation of the final report with an analysis of the selected fragments. The themes were grouped into linked dimensions to provide knowledge. A preliminary analysis was carried out by the first author. The second researcher examined and compared their analyses independently. The quotes presented in the results section were included for their representativeness and selected after their accuracy was verified. The analysis was carried out in Spanish and the quotes later translated into English and reviewed by an accredited native translator not directly involved with the data collection.

We used Lincoln’s and Guba’s criteria to establish the trustworthiness of the study [25]. All data was triangulated by two of the study’s researchers to enhance validity and confirmability of the findings.

### Ethical considerations

We received ethical approval from the Andalusian Biomedical Research Ethics Portal, Ethics review committee (Ref: TFM-IGAL-2020). Verbal informed consent was obtained from all participants prior to the interviews. An information sheet informed the participants about the study procedure, purpose, risks and benefits. Confidentiality of the data was guaranteed in accordance with the Protection of Personal Data and Guarantee of Digital Rights Law and the ethical principles contained in the Declaration of Helsinki and its subsequent modifications [26, 27].

## Results

Initially, from a list of 36 Latinas, 31 participants met the inclusion criteria. The final number of participants interviewed was nineteen: six declined to participate at the last moment and another six either did not have the time to participate or the necessary connectivity for a video call. The total number of participants was based on data saturation. The interviews lasted between 20 and 70 min. The nineteen participants were aged between 22 and 43 years old and came from six different countries in Latin America. Nevertheless, there was heterogeneity of home countries with Peru being the most common (Table 2). Those mothers who had a longer breastfeeding duration were associated with an advanced educational level, multiparity, legal status (legitimate inmigrants) at the time the baby was born, and participating actively in the Breastfeeding Support Group.

**Table 2:** Sociodemographic characteristics

Participants	Age (years)	Education level	Children	Home country	Documentation status	Host country (time)
MML-1	25–35	University	2	Costa Rica	Regularized^a^	9 years
MML −2	25–35	A level	3	Honduras	Regularized	5 years
MML −3	> 35	University	1	Costa Rica	Regularized	20 years
MML −4	> 35	Vocational training	1	Peru	Not regularized	2 months
MML −5	> 35	Postgraduate	2	Peru	Regularized	4 years
MML −6	> 35	A level	4	Peru	Regularized	14 years
MML −7	25–35	Secondary	3	Peru	Not regularized	8 months
MML −8	> 35	University	1	Peru	Regularized	4 years
MML −9	25–35	Vocational training	2	Honduras	Regularized	2 years
MML −10	> 35	Postgraduate	1	Costa Rica	Regularized	2 years
MML −11	25–35	Vocational training	2	Venezuela	Not regularized	1 year
MML −12	> 35	University	2	Panama	Regularized	11 years
MML −13	> 35	Primary	3	Honduras	Regularized	4 years
MML −14	> 35	Vocational training	3	Colombia	Regularized	2 years
MML −15	> 35	Vocational training	4	Venezuela	Regularized	3 years
MML −16	25–35	A level	2	Colombia	Regularized	12 years
MML −17	> 35	Vocational training	1	Peru	Regularized	20 years
MML −18	25–35	University	2	Peru	Regularized	4 years
MML −19	< 25	Secondary	1	Colombia	Regularized	12 years

**Note.** Source: Self-made

^a^Regularized means that the person who came from a third-country and stayed illegally in the European Union is awardeda legal status

During the data analysis, two main categories and ten interrelated sub-categories and their interpretation were identified by the researchers (Table 3).

**Table 3:** Categories and sub-categories

Categories	Sub-categories
Breastfeeding barriers	Work conditions
	Precarious socioeconomic conditions
	Lack of support: health professionals, family and society
	Physiological changes, pain and fatigue
	Ignorance and wrong beliefs
Breastfeeding facilitators	Support networks: partner, health professionals and family
	Host country versus home country
	Religious practices/worship
	Appropriate attitude, knowledge and experience
	Breastfeeding support groups

### Breastfeeding barriers

#### Working conditions

Working conditions were the main obstacle to breastfeeding because there were no places to express and store breastmilk nor was there enough time for pumping while at work.

*“ . . . if they express their milk, where do they store it . . . the majority . . . resort to formula.” (MML-5).*

*“ . . . you have that pressure . . . either you stop working to breastfeed or you continue to work so that you can get food for everyone.” (MML-9)*

#### Precarious socioeconomic conditions

Despite their education and training qualifications from their home countries, the mothers had limited employment options. All said that because of their irregular documentation status they could only access casual employment (cleaning and all forms of health care, particularly care for adults) without paid maternity leave. The bureaucratic challenges for validating degrees and other certifications from their home countries limited access to jobs that reflected their training.

*“ . . . if we don't have documentation . . . who is going to give you your salary while you are on leave? . . . how are we going to cover what we have to pay?” (MML-2)*

#### Lack of support: health professionals, family, and society

The women also identified unpleasant experiences with some healthcare professionals, complaining that professionals played a paternalistic role participants found difficult to trust because they encouraged the women to interrupt breastfeeding. The participants also complained that the knowledge of some health professionals needed to be updated.

*“Health professionals are not trained . . . it is horrible how little they know about breastfeeding . . . he called me negligent . . .” (MML-1)*

Others stated that the extended family could negatively interfere with the breastfeeding process as some offer inadequate or erroneous information that encourages mothers to substitute formula milk for breastmilk.

*“ . . . I was not breastfeeding all the time required and it was because of the inadequate information that I had around me . . . they [family] are trapping you until you switch to formula milk.” (MML-12)*

A further issue identified by the the participants was that they felt questioned and judged by society if they prolonged breastfeeding or decided to, or were forced to, stop breastfeeding early on.

*“But in [Spanish] society, there are many people who see me on the street, with the child… [and they say to her] “Are you still breastfeeding the baby? When are you going to stop? . . . Sometimes, I break down, I feel frustrated . . . nervous because everyone comes and tells you that this is not right [extending breastfeeding for a long time].” (MML-5)*

Finally, one-third of the participants (six women) stated that the aesthetic component of breastfeeding was another barrier imposed by society. However, our participants recognized that biological function prevails over aesthetics.

*“I felt sad and accused . . . because people didn’t want me to breastfeed my children so that my breasts wouldn’t droop.” (MML-9)*

#### Physiological changes, pain, and fatigue

The women emphasized that the most common problems they experienced were ankyloglossia (tongue tied) or nipple abnormalities such as inverted or cracked nipples. These conditions impeded proper latching and required teaching the mothers to effectively breastfeed. Additionally, most mothers admitted that they wanted to stop breastfeeding because of the pain and fatigue they experienced. Although the pain could be intense, it diminishes over time, while maternal tiredness increased due to continued demands.

*“I encountered the cracks, the pain, the frenulum issue . . . that was what prevented my baby from [suckling] well.” (MML-12)*

#### Ignorance and erroneous beliefs

Everyone recognized that insufficient milk greatly hindered breastfeeding in addition to insufficient knowledge such as breast stimulation techniques, posture, or lack of scientific information.

*“ . . . A difficulty that mothers have is the misinformation on . . . unknown topics,* [such as] *tongue-tie, interferences, pacifiers.” (MML-1).*

*“We think that, instinctively, we already do it well [breastfeed], but we don’t. If someone doesn’t explain it to you, then you don’t know how to do it.” (MML-5)*

*“. . . When mothers are inexperienced, they do not know how to breastfeed the baby. So their nipples get sore and that makes them not want to breastfeed.” (MML-14).*

One-third of the mothers also identified incorrect breastfeeding beliefs such as the baby is not satisfied after breastfeeding, and the quality of the breast milk is not good to gain weight, among which the perception of low or insufficient milk supply while breastfeeding predominated.

*“Women are afraid of not having enough milk to give to their babies and that is when they stop breastfeeding and start using formula.” (MML-19)*

### Breastfeeding facilitators

#### Support networks: partner, health professionals, and family

The participants acknowledged their partners as their main source of support. Partners were essential if a mother opted to breastfeed instead of offering formula milk. The fathers also helped to care for the baby and with household chores. Similarly, the participants received support from networks made up of mothers, sisters or close friends.

*“If he [husband] hadn’t made this big effort with me, I probably wouldn’t have been nursing for 25 months.” (MML-3)*

These women also viewed the health professionals who positively influenced them as indispensable to their success with breastfeeding. They identified their midwife as the closest health professional who provided them with knowledge and support while breastfeeding.

*“I really received a good explanation from my midwife [about breastfeeding], she was very hands on”(MML-5)*

#### Host country versus home country

Most participants identified the host country, Spain, as a breastfeeding facilitator. In Spain, they found more institutional resources to support breastfeeding as well as better updated and official information. Conversely, half the participants thought their home country also supported breastfeeding because they had access to family counsel and a greater support network.

*“ . . . there are more institutionalized resources here, such as breastfeeding groups . . . and more informal and traditional information over there.” (MML-10)*

Some participants indicated that differences in breastfeeding duration in Spain were not due to cultural issues. They suggested they reflected individual factors such as lack of access to resources such as breastfeeding support groups, or the circumstances mothers were experiencing at the time.

*“I believe that access to a Breastfeeding Support Group . . . [is] more than a cultural barrier, it’s a barrier because I do not know where to look for help.” (MML-3)*

#### Religious practices/worship

One-third of the women recognized the importance of religion or worship as a source of support during their most difficult moments of breastfeeding. This finding was emphasized by mothers belonging to minority religious groups such as Evangelists or Jehovah’s Witnesses.

“*Emotionally, you feel good, that you can do it . . . people of your same church also give you support and you feel more secure* [Evangelist].” (MML-2)

#### Appropriate attitude, knowledge, and experience

All the participants showed a good attitude towards breastfeeding which they recognized was the best way to feed their babies. Moreover, most attended antenatal classes during pregnancy and showed adequate knowledge about breastfeeding advantages, as well as a satisfactory breastfeeding experiences.

*“It’s tailor-made, like the perfect and exclusive food for your baby . . . there are only advantages.” (MML-10)*

#### Breastfeeding support groups

Longer breastfeeding periods were observed among mothers who actively participated in Breastfeeding Support Groups compared with those who did not. Furthermore, tandem breastfeeding was more frequently observed among mothers who participated in Breastfeeding Support Groups. The participants in Breastfeeding Support Groups had a high educational level, such as university or postgraduate levels, whereas the women who did not get involved in these groups had received vocational training or higher education.

The participants considered it important to increase the number of Breastfeeding Support Groups to help new mothers, with experienced mothers providing knowledge or correcting misconceptions. Mothers also highlighted the various functions of Breastfeeding Support Groups such as offering up-to-date knowledge; psychological support; women’s empowerment; and recreation and social interaction.

*“ . . . it has been a revelation because of the high-quality information . . . I have learned much more with them than from any professional.” (MML-1)*

*“Those little tribes are like my . . . oasis, my relief.” (MML-3)*

*“. . . they give you the chance to meet other people, interact . . . that is the best thing because you come from another country, you don’t know anyone and that helps you a lot.” (MML-15)*

## Discussion

To our knowledge, this study is the first in Spain aiming to understand Latina breatfeeding experiences from a qualitative perspective. In general, our results were consistent with the existing literature [28–30].

In our study, the main element that hindered breastfeeding was paid employment [14, 31–33]. Our participants indicated that the absence of adequate breastfeeding facilities in the workplace together with an early return to work during the postpartum period were significant barriers to breastfeeding. Women maintained that return to the workplace while breastfeeding was determined by the economic pressure and precarious socioeconomic conditions in which they live, as shown in the literature [34]. The participants commented that, despite having valid legal status, obtaining validation of a foreign degree is difficult. Consequently, they were relegated to unstable jobs with inflexible conditions, no maternity leave (in Spain, maternity leave lasts 16 weeks to allow a good breastfeeding and motherhood experience), and long working hours. These conditions limit a mother’s ability to maintain breastfeeding [16, 34–36].

The support the mothers received from healthcare professionals and their family was controversial, as this support was identified as both a facilitator of and barrier to breastfeeding. Participants commented that health professionals acted as a barrier when they had little breastfeeding knowledge because this led to unpleasant situations such as inadequate care and paternalistic responses to doubts or outdated information.

*“Health professionals are not trained, it is horrible how little they know about the subject (breastfeeding) . . . He called me negligent for ignoring him. It is not professional that I, as a mother, know more about breastfeeding than a health worker.*” (MML-1)

*“The first pediatrician who saw my little girl got angry with me because I told him that I was not going to give her formula, only exclusive breastfeeding and he told me: ‘she is going to be malnourished’“* (MML-12) [30, 37, 38]. Thus, it could be concluded that the negative influence of the health professionals could be due to lack of updating or inadequate training in breastfeeding. In that case, they would not know how to respond to mothers’ needs and concerns and so act as breastfeeding barriers. Conversely, when health professionals were adequately trained, the women considered their support as essential for breastfeeding success [29, 31]. In this context, the participants identified their midwives as the health professionals giving them advice best adapted to their needs [12, 14].

Many of the mothers indicated that at times they felt forced to formula feed their infants in order to avoid offensive comments from family members or the public [31]. However, when their partners or family members were pro-breastfeeding they acted as positive reinforcers of the value of continuing to breastfeed [29, 31].

Another facilitator to breastfeeding was the influence of religious beliefs as a motivation to continue breastfeeding and a surce of support during difficult moments while breastfeeding [39, 40].

*“Emotionally, you feel good, that you can do it . . . people of your same church also give you support and you feel more secure [Evangelist].” (MML-2)*

*“I belong to an evangelical church and I know that God is the one who gives me the strength to continue breastfeeding.” (MML-13)*

However, in our study, the above finding predominated among Evangelists or Jehovah’s Witnesses. Moreover, a proactive attitude towards breastfeeding along with a positive breastfeeding experience and adequate knowledge were associated by the mothers with higher breastfeeding rates because they identified benefits for their babies and themselves [16, 37, 41].

The host country’s influence on breastfeeding maintenance was also influential. Most mothers identified the host country as very supportive of breastfeeding through the resources it made available (up-to-date information, support groups, follow-up and classes with the midwife) [18].

*“ . . . there are more institutionalized resources here, such as breastfeeding groups . . . and more informal and traditional information over there.” (MML-10)*

*“*. *.. the information received by the midwife, the doctor and the team that takes care of you when you are pregnant here, yes, it has been better.” (MML-12)*. Cultural influences were also found to have a positive influence with the longest duration of breastfeeding found among the migrant women who had resided in the host country for the longest period [14, 42]. Furthermore, in our study participation in Breastfeeding Support Groups was associated with a longer breastfeeding duration [43, 44]. Mothers who had resided in Spain the longest reported that they were provided with updated and scientific knowledge as well as psychological support that encouraged and empowered them to breastfeed. Similarly, the Breastfeeding Support Group offered them a recreational space that facilitated sociocultural integration [38, 45]_._

Finally, the participants expressed the need for breastfeeding instruction and regular contact with healthcare professionals before and during birth, as well as during the early postpartum period [14, 30, 46]. Breastfeeding Support Groups were also associated with an improvement in breastfeeding duration among Latina mothers [18] with participants demanding greater Breastfeeding Support Group visibility to facilitate access to these groups and ultimately, improve breastfeeding rates in our country. Finally, in common with other studies, participants in the current study emphasised the importance of painful breasts and nipples or fatigue as obstacles to breastfeeding. A lack of breastfeeding knowledge and/or erroneous beliefs such as the perception of insufficient milk were also identified as barriers to breastfeeding [31, 37].

The lockdown that began in March 2020 could be the main limitation of this study, as some mothers may have decided not to take part in the research. Some reported difficulties due to connectivity issues (resources to carry out the interview by video call such via an internet connection, mobile phone, computer or another device with a camera, etc.) or emotional management (mothers afraid that they might be infected with COVID-19 in the workplace (most worked as house cleaners) and talking about the risks intensified their stress. A futher possible limitation of the study is that the interviews were conducted and analysed in Spanish and subsequently translated into English which may have affected the communication of the results [47].

## Conclusions

This study has highlighted the need to promote a more inclusive and culturally sensitive society for migrant groups, such as Latina migrants. In this context, contributing to migrant literature and openness to diversity should be encouraged. We believe that by identifying breastfeeding facilitators we can use the findings to mitigate the negative effect of barriers to breastfeeding. This goal requires researchers to always take into account the cultural needs of the group.

Most of our participants were convinced that breastfeeding was the best option, but needed more information and knowledge to successfully breastfeed. Peer support has been shown to be helpful, and there is a need for health care professionals to adapt breastfeeding-friendly practices to the circumstance of migrants, particuarly barriers confronted in the work place and a lack of support networks. The participants also identified the need for more support from caregivers, sensitivity to cultural diversity, and well-trained health professionals.

## Supplementary Information

**Additional file 1.** COREQ (COnsolidated criteria for REporting Qualitative research) Checklist.

## Data Availability

The datasets used and/or analysed in the current study are available from the corresponding author upon reasonable request.

## Abbreviations

COREQ: Consolidated Criteria for Reporting Qualitative Research
REC: Ethics Review Committee
WHO: World Health Organization
